# Pathogenesis of Huntington’s Disease: An Emphasis on Molecular Pathways and Prevention by Natural Remedies

**DOI:** 10.3390/brainsci12101389

**Published:** 2022-10-14

**Authors:** Zainab Irfan, Sofia Khanam, Varnita Karmakar, Sayeed Mohammed Firdous, Bothaina Samih Ismail Abou El Khier, Ilyas Khan, Muneeb U. Rehman, Andleeb Khan

**Affiliations:** 1 Department of Pharmaceutical Technology, Brainware University, Kolkata 700125, West Bengal, India; 2 Department of Pharmacology, Calcutta Institute of Pharmaceutical Technology & AHS, Howrah 711316, West Bengal, India; 3 Department of Pharmacology, Eminent College of Pharmaceutical Technology, Barasat 700126, West Bengal, India; 4 Architectural Engineering Department, Faculty of Engineering and Technology, Future University in Egypt, New Cairo 11835, Egypt; 5 Department of Mathematics, College of Science Al-Zulfi, Majmaah University, Al-Majmaah 11952, Saudi Arabia; 6 Department of Clinical Pharmacy, College of Pharmacy, King Saud University, Riyadh 11451, Saudi Arabia; 7 Department of Pharmacology and Toxicology, College of Pharmacy, Jazan University, Jazan 45142, Saudi Arabia

**Keywords:** Huntington’s disease (HD), neurodegenerative disorder, pathogenesis, huntingtin (*htt*), natural drugs, CAG expansion, natural products

## Abstract

Background: Huntington’s disease is an inherited autosomal dominant trait neuro-degenerative disorder caused by changes (mutations) of a gene called huntingtin (*htt*) that is located on the short arm (p) of chromosome 4, CAG expansion mutation. It is characterized by unusual movements, cognitive and psychiatric disorders. Objective: This review was undertaken to apprehend biological pathways of Huntington’s disease (HD) pathogenesis and its management by nature-derived products. Natural products can be lucrative for the management of HD as it shows protection against HD in pre-clinical trials. Advanced research is still required to assess the therapeutic effectiveness of the known organic products and their isolated compounds in HD experimental models. Summary: Degeneration of neurons in Huntington’s disease is distinguished by progressive loss of motor coordination and muscle function. This is due to the expansion of CAG trinucleotide in the first exon of the *htt* gene responsible for neuronal death and neuronal network degeneration in the brain. It is believed that the factors such as molecular genetics, oxidative stress, excitotoxicity, mitochondrial dysfunction, neuroglia dysfunction, protein aggregation, and altered UPS leads to HD. The defensive effect of the natural product provides therapeutic efficacy against HD. Recent reports on natural drugs have enlightened the protective role against HD via antioxidant, anti-inflammatory, antiapoptotic, and neurofunctional regulation.

## 1. Introduction

Huntington’s disease (HD) is an autosomal, escalating, and dominantly-inherited disease caused due to degeneration of neurons characterized by impairment of choreatic movements, and behavioral and psychiatric loss, principally in the cerebral cortex and striatum [1,2,3]. It claims its name from a physician, George Huntington, who first described the illness in 1872 [4]. In 1974, the first publication on HD was published [5]. HD is the earliest genetic condition to be linked to a specific chromosome site, and it has a special place in medical genetics research [6,7,8,9].

The biological pathways causing the disease are unknown and complicated, even though HD is distinguished by a well-defined genetic origin. Different mechanisms such as molecular genetics, leading to oxidative stress, metabolic dysfunction, and mitochondrial dysfunction, explain the pathological process of HD [10,11]. Thus, early diagnosis of clinical manifestation along with meticulous management becomes crucial.

HD is a neurological disease that develops slowly and is triggered due to sudden changes in the HD protein huntingtin (*htt*) [12,13]. An extension within the CAG repeat tract causes a mutation in *htt*, resulting in longer lengths of polyglutamine (polyQ) in the encoded protein. In unaffected populations, wild-type alleles contain up to 35 CAG repetitions, whereas 36 or more CAG repetitions specify HD allele [12,14,15,16]. The total amount of CAG repeats, as well as the phase of appearance of symptoms, is inversely connected, i.e., larger CAG repeat extensions are related to an initial phase of onset [17,18,19]. The protein encoded by the *htt* gene is a 348-kDa multidomain protein with a polymorphic glutamine/proline-rich domain at its amino terminus [20,21]. HD is currently the most extensively reported hereditary neurodegenerative disease that has diagnostic and prognostic genetic testing, with the probability of gene-targeted therapy in the nearby future [6]. HD was among the first diseases to be genetically tested before birth. Certainly, neuroimaging techniques have provided predictive and diagnostic genetic screening for ailment identification and its implications on sick persons and families through specialized facilities and genetic testing procedures [6,22,23].

Specific neurodevelopmental findings in HD include striatal degeneration and neuronal death, notably in the caudate nuclei, which target the cerebral cortex, pallidum, thalamus, brainstem, and cerebellum [24]. Neuroinflammation and microglial activation are hallmarks of the preliminary phase of HD [25]. In HD plasma, the levels of IL-6, matrix metallopeptidase 9, vascular endothelial growth factor (VEGF), and TGF-1 were significantly elevated, whilst the levels of IL-18 were markedly decreased. The prevalence of HD was reversely linked with plasma IL-6 [26]. HD is a severe autosomal-dominant late-onset neurological disorder that leads to chronic and incurable motor defects, leading to difficulties with coordination and mobility in addition to psychological-behavioral abnormalities. One of HD’s complications is visual deficit [27].

Patients with HD have been shown to exhibit important visual system impairments, notably retinal thinning, thinning of the temporal retinal nerve fibre layer, deletion of retinal ganglion cells, vision impairment elicited possibilities, poor colour vision, and poor motion perception [28,29,30,31]. When the retina of transgenic HD R6/1 mice was examined under an electron microscope, it was discovered that the diseased retina of HD mice is loaded with peculiar organelles called myelinosomes. Myelinosomes, which contain mutant m*HTT*-exon 1, can be released from glial Müller cells and then integrated into neuronal cells via a membrane fusion mechanism, aiding in the spread of HD [32].

The CNS has demonstrated the activation of the neurotoxic branch of the Kynurenine Pathway. The serum of HD patients had lower tryptophan (TRP), greater amounts of Kynurenine (KYN), and higher KYN/TRP ratios, which suggested stronger Indolamine 2,3-dioxygenase activity [33,34]. The intensity of symptoms and the quantity of CAG repeats were inversely connected with the levels of TRP. At the same time, the inflammatory condition was positively correlated with the levels of Anthranilic acid, which may be a useful biomarker [35].

The objective of the therapy is to reduce symptoms and improve the standard of life [36]. For symptomatic care, there are numerous effective choices; however, both drug-based and non-drug-based therapy is used [37]. Nature, as the best combinatorial chemist and home to hundreds of plant species, could have a direct medicinal influence on the body [5,38]. Natural ingredients with antioxidants, anti-inflammation, anti-apoptosis, calcium antagonization, and neurofunctional regulatory properties have been proven to cure or treat neurodegenerative diseases [39].

A few of the significant phytochemicals having neuroprotective activity include flavonoids, celastrol, sesamol, and trehalose [10,40]. Drug-based therapy involves symptomatic treatment through various therapeutic drugs [41]. HD therapeutic development and advancement that modulates the m*HTT* level through genetic transfer is one of the most important strategies to ameliorate the disease [42].

Here, we review to accentuate the significance of the underlying biological procedure intricate in the pathological process of HD. Moreover, an effort has been taken to enlighten different diagnostic techniques and various natural drugs and their phytochemicals with valuable effects against HD. Valuable insights provided by this review on HD increase hope for more definitive therapeutic strategies.

## 2. Methodology

A Medline (PubMed), Cochrane Library, and Embase-based literature survey were performed using keywords of “neurodegenerative diseases, prevention, non-pharmacological therapies, and neurodegenerative diseases, phytochemicals, Huntington’s till July 2022.

Two independent authors screened all the titles and abstracts of the retrieved data, and disagreements were resolved by the consensus of a third author. Duplicated entries, retracted publications, studies on other diseases or conditions different from NDs or its subtypes, studies without statistical analysis, non-English written papers, publications that are not research studies (i.e., commentaries, letters, editorials, reviews, and meta-analysis), and any other article that did not fit within the scope of this review were excluded. Articles listed in the references were also reviewed in search of more data.

A total of 550 results were retrieved and screened with the above keywords. Of these, 249 publications were selected and eventually used for qualitative analysis.

## 3. Epidemiology

Between diverse geographic regions, the universality of HD spreads more than ten folds [43,44]. HD is a neuropsychiatric disorder with pervasiveness of 5–10 people per one lakh in the white-skinned European-origin community. In Japan, the incidence is around one-tenth of the white-skinned of European origin population [45,46]. There is no gender predominance, and its prevalence is 5–8 per 100,000 individuals worldwide. The highest frequency of HD is found in European countries. The universality of HD ranges from 4.1 to 8.4 per lakh people estimated in the USA [47]. In India, the universality of HD is greater and similar to that seen in Western Europe [48].

## 4. Clinical Assessment

HD is associated with dysfunction in motor, cognitive, and psychiatric functions, symptoms of which are tabulated in Table 1. People are suffering from HD show specific and characteristic cognitive difficulties [49]. Traditionally, this cognitive change has been stated to as dementia. Changes in behavior are a distinctive characteristic of HD, which is the most stressful facet of the circumstance for families and individuals coping with it [44,50]. Other less eminent but weakening characteristics of HD comprise unexpected weight loss, sleep, circadian rhythm problems, and autonomic nervous system (ANS) dysfunction. The average life span of the start of the disorder is 30 to 50 years old, with a range of 2 to 85 years, and the disease lasts 17 to 20 years on average [1,51].

## 5. Developmental Stages of HD

HD can be categorized into five stages (Table 2). Individuals with early-stage HD are functional. They continue to work, manage expenses, drive, and live independently. Individuals in the middle stage of HD begin to lose their power to work or drive [63]. They cannot handle their finances or conduct household tasks, but they can eat, dress, and keep themselves clean with assistance. It becomes difficult to handle patients suffering from HD as they are unable to sequence, consolidate, or prioritize information. As the stage moves into the early advanced and advanced stage of HD, individuals need complete assistance for daily living. Although they are bedridden and non-verbal, still people with HD seem to recollect some comprehension [64]. The total functional capacity is the most widely used rating scale based on functional abilities. A rating scale that rates the person’s level of independence based on occupation, the capability to handle finance, and the capability to perform household chores [59].

## 6. Huntington’s Pathogenesis: Mechanistic and Genetic Approach

HD is generated by a mutation in the *htt* gene, which codes for huntingtin, a ubiquitously expressed protein with 36 or more CAG repeats [12,14]. Despite the information that the genetic origin of HD is well documented, the various molecular modifications investigated in HD are broad and not utterly understood [12]. The expression of an enlarged PolyQ contributes to the impairment of wild-type protein [68]. Consequently, wild-type huntingtin loss or inactivation causes neurodegeneration. Different pathways involved in HD are illustrated below.

### 6.1. Transcriptional Dysregulation

One of the major players in the pathogenic process of HD is attributed to transcriptional dysregulation [69]. Mutant *htt* impairs transcription, according to DNA microarray studies [70,71]. This mutant *htt* interferes with transcription regulators such as p53, CREB–binding protein (CBP), and cAMP response element-binding (CREB) protein, all of which are significant for cellular functions and survival [72,73,74,75,76]. Dysregulation of CREB and Neuron restrictive silencer elements (NRSE) mediated transcription in HD and in normal person are described below.

#### 6.1.1. cAMP Response Element-Binding (CREB) Protein Pathway

##### Normal Individual

**Figure 1:**
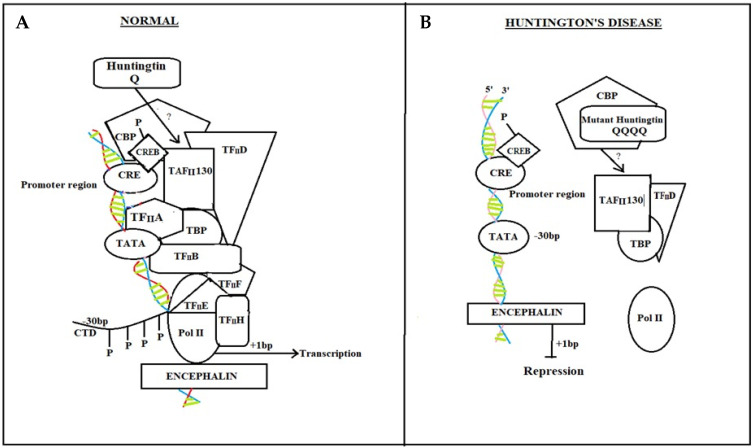
CREB protein pathway in Normal individual and HD diseased patient. In normal individuals, CREB binding with CRE enables normal neuronal responses by activating a cascade of transcriptional factors (**A**) while in Huntington’s disease patients, due to mutant *htt* gene, CRE transcriptional cascade breaks, and there is no attachment of CBP and TAFII 130 with CRE. Pol II dispositioned (**B**).

In normal individuals (Figure 1A), the stimulation of certain transcription factors, such as CREB, which binds to DNA regions containing CRE in cellular promoters, is significant for neuronal survival [77]. Transcriptional activation due to CREB phosphorylation allows the recruitment of CBP [78]. CBP remodels chromatin further, allowing CREB to engage the TAFII130 component of TFIID [79]. Thus, the overall transcriptional machinery, including transcriptional factors TFIIA, B, D, E, F, and H, and TATA-binding protein (TBP), are activated. TFIIH phosphorylates RNA polymerase II (Pol II) in its carboxy-terminal domain (CTD) to commence transcription once it is suitable [12,80,81].

##### HD Diseased Patient

Mutant *htt* interrupts CRE-mediated transcription in HD patients (Figure 1B) with direct interaction or sequestration of CBP and TAFII130 in the nucleus. CBP and TAFII 130 eventually lose their capability to attach to CRE sites in cellular promoters. Because the general transcription machinery, as well as Pol II, is not adequately positioned to the promoter, transcriptional activation is hampered [12,82,83].

#### 6.1.2. NRSE Mediated Pathway

##### Normal Individual

**Figure 2:**
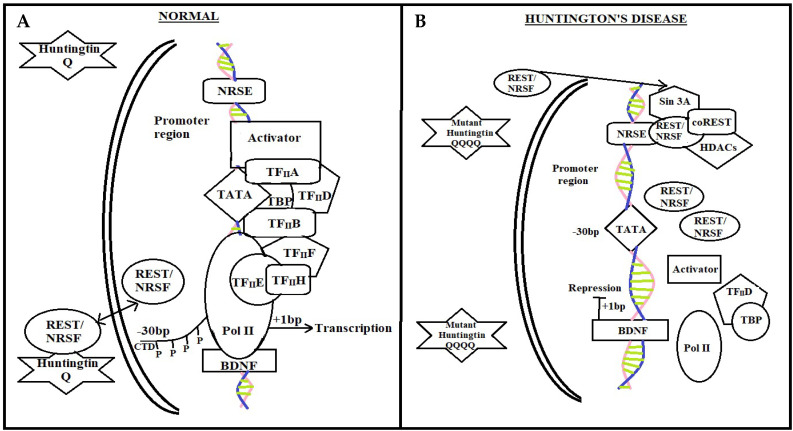
NRSE mediated pathway in a normal individual and diseased patient. In normal individuals, transcription factor REST–NRSF binds to NRSEs in neuronal gene promoters such as in the brain-derived neurotrophic factor (BDNF) gene. By interacting with REST-NRSF in the cytoplasm and lowering its availability in the nucleus to bind to NRSE sites, wild-type *htt* maintains BDNF synthesis, which is a crucial survival factor for the striatal neurons that die in HD. In these circumstances, activators can bind to the BDNF promoter regions and then recruit the general transcriptional machinery and Pol II, promoting the transcription of BDNF. While in HD, REST-NRSF levels in the nucleus rise as a result of mutant *htt*’s failure to connect with REST-NRSF in the cytoplasm. In these circumstances, REST-NRSF binds to the NRSE with vigor and stimulates the recruitment of Sin3A-histone-deacetylase complexes (HDACs), which contain histone deacetylase activity for remodeling chromatin into a closed architecture and squelching BDNF transcription. REST stands for repressor-element-1 transcription factor. NRSE stands for neuron-restrictive silencer element.

In a normal individual (Figure 2A), the wild type *htt* regulates the action of the genes that contain (NRSE) by modifying cytoplasmic hiring to the nucleus of NRSE binding transcription factors, which are critical survival factors for striatal neurons [84]. The transcription factor REST-NRSF (repressor-element-1 transcription factor-neuron restrictive silencer factor) attaches to NRSEs in neuronal gene promoters such as the BDNF gene [85]. Wild type *htt* survives BDNF synthesis by networking with REST-NRSF in the cytoplasm, alleviating its capacity to attach to NRSE sites in the nucleus. Activators that attach to the BDNF promoter regions and afterward engage the transcription process and pol II boost BDNF transcription in these settings [12,86,87].

##### Diseased HD Patient

In HD individuals, mutant *htt* decline to interlink with REST–NRSF, resulting in elevated REST–NRSF levels in the nucleus (Figure 2B). REST–NRSF attaches to the NRSE with vigor in these conditions, promoting the incorporation of Sin3A–histone deacetylase complexes (HDACs) with histone deacetylase activity for chromatin remodeling framework [88,89,90]. Consequently, the expression of NRSE, NRSF, BDNF, and REST are suppressed [12].

### 6.2. Ubiquitin-Protease System

#### 6.2.1. Chaperones and the Proteasome Dysfunction

Through numerous sequential actions, molecular chaperones are used in the precise folding of newly produced proteins into correct conformation, and this procedure can be fruitless [91]. Heat shock proteins play an important role in protein folding and quality control. In the context of polyglutamine diseases, such as HD, heat shock protein 70 (Hsp70; Hspa1a/b), Hsp40 (Dnajb1), and Hsp90 (Hsp90aa1 and Hsp90ab1) have been the subject of several studies. Elevation of Hsp70 levels has been found to be neuroprotective in several animal models [92]. For instance, Hsp70 overexpression suppressed neuropathology and improved motor function in a spinocerebellar ataxia mouse model. Pharmacological and genetic Hsp90 inhibition induces m*Htt* degradation. Furthermore, Hsp70 and Hsp40 attenuated the assembly of polyglutamine proteins into amyloid-like fibrils [93]. As a result, proteins must either be refolded into their proper configuration, or the ubiquitin-proteasome system (UPS) degrades them [94]. Hsp70 (Heat-shock protein 70) and Hsp40 (Heat-shock protein 40) are two prominent types of molecular chaperones that help in the folding of polypeptides and hence prevent misfolded proteins development. Furthermore, in other polyQ proteins, the mutant *htt* has been shown to be linked with the Hsp70 and Hsp40 chaperone families and also colocalize with aggregates [95]. Chaperone sequestration into aggregates reduces the quantity of soluble chaperones in the cell, which helps to alleviate aberrant protein folding [96].

#### 6.2.2. Ubiquitin-Proteasome System Impairment

**Figure 3:**
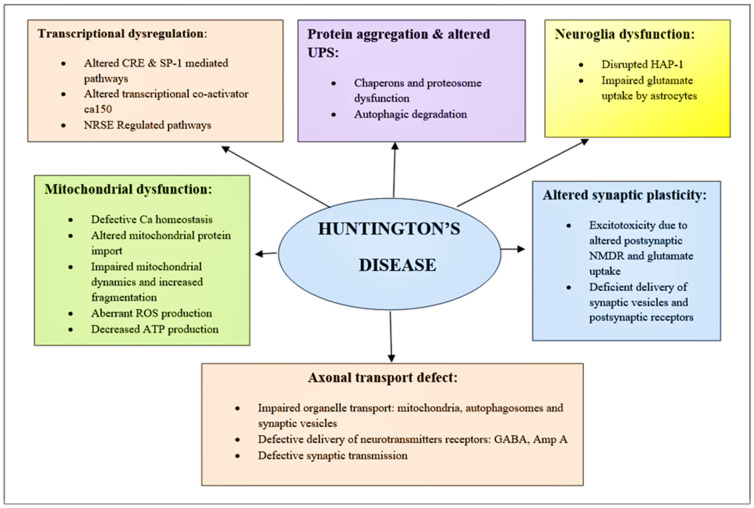
Schematic of diverging pathways leading to the pathogenesis of HD. Here is the mechanism of pathogenesis, paying particular attention to those that are related to promising therapeutic targets. BDNF, Brain-derived neurotrophic factor; ROS, reactive oxygen species; NMDAR, N-methyl-D-aspartate receptor; UPS, Ubiquitin-protease System; NRSE, Neuron restrictive silencer elements; CRE, cAMP response element.

The stages involved in the UPS system failing and causing cellular pathogenesis in HD (Figure 3) are listed below [12].

**Figure 4:**
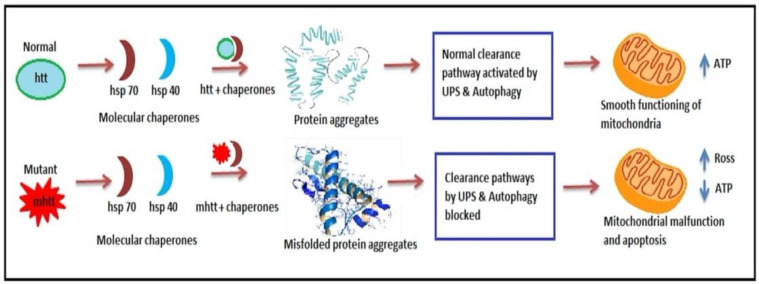
Ubiquitin-proteasome impairment in HD. In Normal individuals, Hsp70 and Hsp40 aid in transcriptional functions resulting in protein formation and aggregation and smooth functioning of mitochondria. However, in HD diseased patient mutant mhtt causes misfolding of protein aggregates and disrupts clearance pathways leading to mitochondrial dysfunction.

(1) Hsp70 and Hsp40, two molecular chaperones, cause newly produced *htt* to pleat into a native structure [97]. The cytoplasmic functions of wild-type *htt* include vesicle transport, clathrin-mediated endocytosis, cytoskeletal anchoring, postsynaptic signaling, and neuronal transport. On the other hand, this *htt* might be carried into the nucleus and aid in transcriptional control.
(2) Chaperones aid in the identification of aberrant proteins, promoting refolding or Ub (ubiquitination) and obliteration by the 26S proteasome [98]. Mutations produce conformational anomalies and improper folding of *htt* in HD patients, resulting in a buildup of misfolded *htt* in the cytoplasm if chaperones are not precise.
(3) However, mutant *htt* is cleaved by proteases, resulting in the formation of amino-terminal components that form β-sheet structures [99].
(4) As a result, cleaved N-terminal fragments or mutant full-length *htt* cause toxicity, forming soluble monomers, oligomers, or massive insoluble aggregates. Mutant forms of *htt* in cytoplasm disrupt the UPS, allowing misfolded proteins to accumulate [100].
(5) Vesicle transport and clathrin-mediated endocytosis are disrupted by these noxious proteins. Furthermore, mutant *htt* promotes pro-apoptotic proteins through mitochondrial malfunction, causing cellular noxiousness and other negative implications [101].
(6) For defence, the cell gathers hazardous pieces into ubiquitinated cytoplasmic perinuclear aggregates [102].
(7) Mutant *htt* are translocated into the nucleus, resulting in nuclear inclusions that can interrupt transcription and the UPS (Figure 4) [103].

#### 6.2.3. Altered Synaptic Plasticity

Early pathogenic processes in HD include synaptic and neuronal anomalies [104]. Reduced transcription of significant genes in signaling and neurotransmission disrupts neuronal homeostasis [105]. This causes imperfection accompanied by their axons in the transmission of organelles and proteins. Pathogenic *htt* also prevents organelle transportation across the axon [106,107]. *Htt* accelerates vesicle trafficking by acting as a scaffold connecting microtubules, cargoes, and motor proteins including dyneins and kinesins [108,109]. Huntingtin-associated protein1 (HAP1) mediates this interface, which tends to be impaired in HD disease [110].

#### 6.2.4. Mitochondrial Dysfunction

Mitochondria serve as locations for oxidative phosphorylation and cellular respiration, both of which result in the production of ATP. They are also important for keeping the cytosol’s calcium concentration low. A mitochondrial defect has been discovered in HD patients, which results in lower mitochondrial oxygen consumption, glucose metabolism, and cAMP levels in the cerebrospinal fluid [111,112,113]. Oxidative stress is also responsible for the neurodegenerative procedure of HD. Since mitochondria are the primary communicator of ROS (reactive oxygen species) in neurons, oxidative stress is related to mitochondrial dysfunction in HD. With an enhancement in ROS or RNS (reactive nitrogen species) production, susceptible neurons in the patient’s brain suffering from HD may be unable to handle it well. Increase in levels of ROS or RNS in membranes may boost an intracellular cascade of oxidative stress by triggering lipid peroxidation and oxidizing DNA and proteins [114,115]. A substantial rise of 8-hydroxydeoxyguanosine, an oxidized DNA marker, and a higher surge of malondialdehyde (MDA), lipid peroxidation marker is perceived in the brain of HD. Oxidative stress also promotes mutant *htt*-dependent cell death by mimicking proteasomal malfunction and *htt* aggregation. Elevation of free radical outcomes in the impairment of mitochondrial function, metabolic dysfunction, impairment of energy production, and excitotoxicity [116,117,118,119,120,121].

### 6.3. Neuroglia Dysfunction

#### 6.3.1. Astrocytes and Microglial Dysfunction

*Htt* is known to be much more abundant in neurons as compared to non-neuronal glial cells. Glial cells are majorly responsible for HD progression and pathogenesis [122,123]. Astrocytes, a kind of glia, protect neurons from excitotoxicity by providing support and allowing extracellular glutamate absorption. In the occurrence of HD disease, however, an N-terminal *htt* with 160Q is expressed exclusively in astrocytes [124]. Consequently, HD astrocytes contribute to neurological symptoms as well as other problems, such as reduced chemokine CCL5 or BDNF discharge [125].

Microglial and astrocytic contributes to neuronal death in HD. Surveilling microglia are activated by stimulating molecules through NF-κB signaling, upregulation of PU1, and CCAT binding. Activated microglia and reactive astrocytes produce ROS and neurotoxic molecules (such as quinolinic acid), which can induce molecular processes leading to neuronal death. Stimulatory molecules also induce reactive astrogliosis that leads to the upregulation of pro-inflammatory cytokine production, glutamate excitotoxicity, and hyperexcitability of neurons. Activated microglia can adopt different states, commonly, this polarization has been categorized as M1 and M2 states, and microglial cells can alternate between the two states. M1 microglia role in the inflammatory response and are thought to be the major initiators of both innate and adaptive immunity in the brain [126]. These cells have a phagocytic function and will release cytotoxic factors such as nitric oxide (NO), ROS, and quinolinic acid to confer toxicity to invading pathogens [126,127]. M2 microglia also carry out phagocytosis but contrary to the role of M1 microglia, M2 microglia exhibit an anti-inflammatory role [126]. This is through the release of anti-inflammatory mediators such as interleukin 4 (IL-4), interleukin 13 (IL-13), IL-10, and transforming growth factor beta (TGF-β) to suppress inflammatory responses [128].

#### 6.3.2. Release of Pro-Inflammatory Cytokines and Chemokines

The secretion of pro-inflammatory cytokines by astrocytes is associated with neuroinflammation and neurodegeneration in HD [41]. Microglial generation of pro-inflammatory cytokines is induced by *htt* expression in immune cells [129]. Mutant *htt* affects inflammatory responses in the peripheral immune system by inhibiting NF-κB signalling, implying that neuro-inflammation is both a reactive and proactive mechanism in disease development [130]. IL-6 is upregulated in the plasma of HD patients [131]. IL-6 stimulates the expression of another acute phase protein: -macroglobulin (M). M is upregulated in plasma of HD patients, mainly in reactive astrocytes, and therefore influences immune proteins and cytokines. Moreover, several mouse models of HD display significantly higher levels of IL-1*β*. IL-1*β* itself is able to directly induce neurotoxicity via activation of tyrosine kinases and phosphorylation of NMDA receptors involving the NF-kappa(κ) B pathway. Inflammatory responses are initiated by different receptors, among others, including the Toll-like receptors (TLRs). TLR activation evokes NF-κB activation resulting in increased transcription of proinflammatory cytokines [132].

### 6.4. Axonal Transport Defect

#### 6.4.1. Defective Synaptic Transmission

Axonal transport is necessary for appropriate transfer to neuronal membranes in order to facilitate synaptic communication. Synaptic excitability in HD is inhibited by a failure of receptor delivery, either GABA_A_ (γ-aminobutyric acid type A) or AMPA (α-amino-3-hydroxy-5-methyl-4-isoxazole propionic acid) receptors [41]. HAP1 connects these receptors to the kinesin motor factor KIF5, and mutant *htt* disrupts this interface [133,134]. Mutant *htt* also prevents the release of BDNF and cortical transport and the regressive transport of its receptor TrkB in the striatum, which is essential to trigger survival signals in the cell body [86,135].

#### 6.4.2. Excitotoxicity and Medium Spiny Neurons (MSNs) Degeneration

In HD, MSNs in the striatum show the most noticeable deterioration [136]. MSNs were also found to be selectively influenced by glutamatergic signals, which facilitate abnormal firing and neurotransmission, NMDA receptor-mediated glutamate activation, and can cause striatal neuronal death via a process known as excitotoxicity [135,137]. Moreover, mutations in HD modify not only NMDAR trafficking in striatal neurons but also the balance of synaptic (pro-survival) and extrasynaptic (detrimental) NMDAR activity [138,139,140,141].

**Figure 5:**
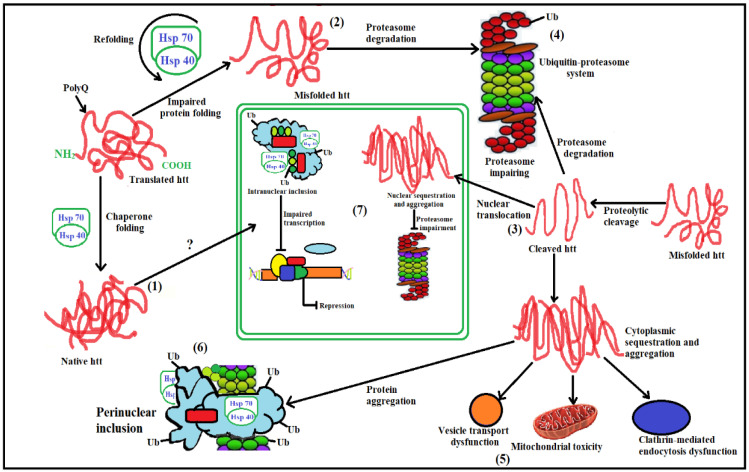
Steps in cellular pathogenesis of HD. (**1**). Huntingtin (*htt*), a freshly generated protein, is encouraged to fold into a native shape by the molecular chaperones Hsp70 and Hsp40. Wild-type *htt* is primarily cytoplasmic and likely participates in postsynaptic signaling, clathrin-mediated endocytosis, vesicle transport, cytoskeletal anchoring, or neuronal transport. *HTT* might enter the nucleus and influence the control of transcription; (**2**). In order to promote either their refolding or their ubiquitination (Ub) and subsequent demise by the 26S proteasome, chaperones can help recognize aberrant proteins. If chaperones are not present to rectify the incorrect folding of *htt* caused by the HD mutation, misfolded *htt* will accumulate in the cytoplasm. The HD mutation causes conformational alterations; (**3**). Alternately, mutant *htt* may also be cleaved by proteolysis, resulting in amino-terminal fragments that produce β-sheet structures; (**4**). Finally, cleaved N-terminal fragments, which may form soluble monomers, oligomers, or huge insoluble aggregates, or full-length mutant *htt* may cause toxicity. Mutant versions of *htt* may damage the ubiquitin-proteasome system (UPS) in the cytoplasm, causing a buildup of more improperly folded proteins; (**5**). These harmful proteins may also interfere with clathrin-mediated endocytosis and regular vesicle transport. Additionally, the presence of mutant *htt* may cause mitochondrial damage, which would directly or indirectly activate pro-apoptotic proteins and increase cellular toxicity as well as other negative effects; (**6**). The cell gathers harmful pieces into ubiquitinated cytoplasmic perinuclear aggregates as a form of self-defense; (**7**). Furthermore, mutant *htt* can go into the nucleus and create nuclear inclusions, which can interfere with transcription and the UPS.

Consequently, several cellular pathways are involved in the evolution of HD (Figure 5).

## 7. Diagnosis

**Figure 6:**
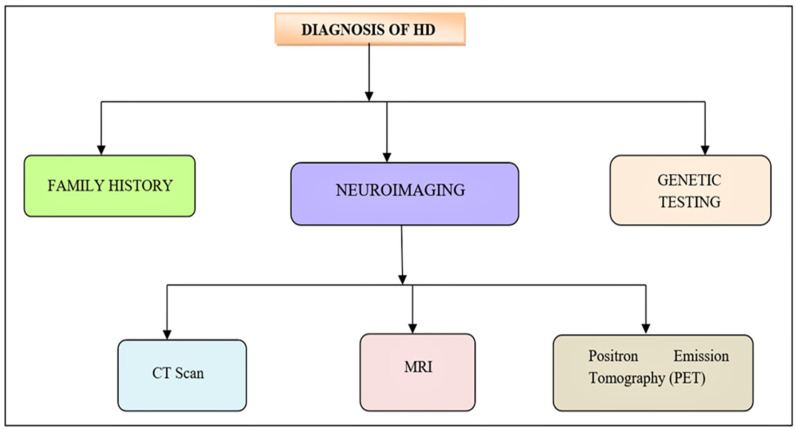
Diagnosis of HD. The initial clinical manifestation of HD is similar to the neurological diseases. Neurological test, definitive genetic examination along with foremost family history is recommended for the diagnosis of HD.

The clinical manifestation of a person with proven HD is utilized to make a diagnosis. To begin, it is essential to obtain an absolute history of the person, which takes precedence over full family history. With or without cognitive or psychiatric changes, motor alterations remain the key clinical criteria [142]. The three main indications, also family history, are required for diagnosis. For all diagnostic tests, the patient must give informed consent [1] (Figure 6). In a patient with chorea, a diagnosis of HD is strongly suspected. The following is an alternative way of diagnosing HD:

(1) Neuroimaging can assist confirm a diagnosis while ruling out other possibilities. A CT scan or an MRI can reveal cerebral atrophy or atrophy of the caudate nucleus. PET (Positron emission tomography) can also show diminution in striatal metabolic rate [143,144].
(2) Genetic testing is a reliable technique to verify a suspected clinical diagnosis [145,146].

## 8. Herbal Management of HD

There have been numerous reports of herbal plants used for the management of HD. Natural constituents derived from herbal plants that have antioxidant, anti-inflammation, anti-apoptosis, immune-stimulatory, calcium antagonization, and neuroprotective properties have been shown to cure or treat HD [39,146,147,148]. Several plants and phytochemicals that have been shown to have anti-HD properties are briefly described.

### 8.1. Panax Ginseng

Ginseng is the dried root of the plant genus *Panax* [149]. Panax ginseng is derived from the Greek words “pan” meaning all and “axos” meaning cure [150]. It is native to Korea and China but is now globally popular and cultivated in countries such as Japan, US, Russia, Canada, Vietnam, Nepal, and Eastern Himalayas [151,152].

Ginseng extract improves neurological and psychological symptoms along with cognitive functions in healthy [153,154,155]. Ginseng has a beneficial effect on psychological feature performance owing to its action on the hippocampal brain [156]. Inhibition of Ca^2+^ entry through glutamate receptors, ginsenosides Rb1 and Rg3, safeguard cortical neurons from glutamate-induced cell damage [157,158]. The inhibition of both NMDA and glutamate-induced by saponins from ginseng increases Ca^2+^ entry through glutamate receptors [159]. An in vitro HD assay with MSN cultures for the investigation of the neuroprotective potential of compounds of ginseng total saponins exhibits neuroprotection by Rb1, Rg5, and Rc are interlinked with the capacity to prevent glutamate induced Ca^2+^ responses. These results can be credited to the active therapeutic choice to treat HD [160]. Ginsenosides Rd, Rb1, and Rb3 have also been shown to protect striatal neuronal damage caused by 3-NP [161]. Active components of ginseng demonstrate to possess beneficial potential such as antioxidant [160], anti-apoptotic [160], anti-inflammatory [161], and immune-stimulatory activities [162]. Moreover, it decreases lipid peroxidation and Ca^2+^ influx and suppresses neuronal excitotoxicity, stabilizes ATP levels in cells, protects neuronal structural integrity, and improves cognitive function [163,164].

### 8.2. Bacopa monnieri

It is a perennial, creeping herb of the family Scrophulariaceae [163,165]. It is also known as Brahmi and is found in warm wetlands [165]. They are indigenous to India and Australia [166]. It is also grown in Sri Lanka, Nepal, China, Vietnam, and Taiwan, Florida, and other southern states of the USA [167].

*B. monnieri* contains dammarane-type triterpenoid saponins, Bacosides A and Bacosides B, which are biomarkers for this species [165,167,168,169]. It also contains different kinds of saponin, including A–G [170,171,172] together with pseudojujubogenin or jujubogenin moieties as aglycones [173], Bacopaside I–V, X, and N_1_ and N_2_ [174,175,176]. The existence of several active constituents such as saponin, alkaloids, sterols, and flavonoids, are attributed to the pharmacological effects of *B. monnieri* [165,177,178]. This plant has potential activity as a memory booster, anti-inflammatory, analgesic, hepatoprotective, and antipyretic, free radical scavenging neuropharmacological disorders such as insomnia and antidepressant agent [179,180]. Bacoside A is the chief constituent for improving memory [181,182]. Due to mechanisms including metal ions complex chelation and enhanced antioxidant defense enzymes enhance the neuroprotective and memory-boosting effects of *B. monneiri* extracts [179,183,184]. An ethanolic extract of *B. monneiri* inactivates 3-nitropropionic acid (NP)-induces dysfunction of mitochondria by altering antioxidant mechanism [185]. 3-NP inactivates the succinate dehydrogenase cell enzyme (SDH) and the electron transport chain complex II–III [186]. It also reduces ROS, malondialdehyde (MDA), and free fatty acid levels [187]. The oral intake of BM’s leaf powder is reported to reduce basal concentrations of several oxidative markers and improve thiol-related antioxidant molecules, and antioxidant enzyme activity [188]. The dietary *B. monneiri* supplements lead to consequential defense against oxidative impairment in the brain along with a defensive effect against neuronal dysfunction due to stress. Thus, *B. monneiri* can be very beneficial in HD treatment [189].

### 8.3. Curcuma longa

*Curcuma longa* is generically called turmeric, a perennial plant [190] with yellow flower [191] dried rhizome of *C. longa* Linn (*Curcuma domestica* Valeton) [150] belonging to Zingiberaceae [190]. It is also cultivated expansively in Malaysia, Bangladesh, Cambodia, China, Indonesia, Thailand, and the Philippines [192,193].

Curcumin, obtained from the rhizome of *C. longa* Linn [194], is a natural agent with several functions and is pharmacologically safe [195]. Curcumin is a phytochemical and a crucial bioactive ingredient that is stated to possess antioxidant, antiangiogenic, anti-inflammatory, antimutagenic, antibacterial, and antiplatelet aggregation potential due to its chemical structure [196].

Curcumin may be effective in the treatment of some ailments characterized by the accumulation of fibrillar protein deposits [197]. Especially under a neurodegenerative condition such as HD, the accumulation of abnormal forms of particular proteins, such as *htt*, may have a role in disease development [198]. A study showed improvement in HD-like neurodegeneration when treated with solid lipid nanoparticles of curcumin (C-SLNs) [199]. There is also a considerable increase in mitochondrial complex activity and cytochrome levels. C-SLNs significantly reduced protein carbonyl production, lipid peroxidation, ROS levels, and mitochondrial swelling by restoring levels of glutathione and superoxide dismutase (SOD) activity [200]. Moreover, treatment with curcumin is reported to enhance cognitive and motor performances, restore succinate dehydrogenase action, and reduce oxidative stress, which inhibits the 3-NP in HD [201]. Curcumin also rescues down-regulated molecular chaperones in HD, including Hsp40 and Hsp70 [202]. Curcumin therapy also restored down-regulated BDNF in HD patients [203,204].

### 8.4. Ginkgo biloba

*Ginkgo biloba*, also called ginkgo, is the deciduous gymnosperm tree in the division Ginkgophyta which belongs to the Ginkgoaceae family [38,205]. Ginkgo is indigenous to Japan, China, Korea, North America, and Europe [150]. *G. biloba*, alike most plant medicines, contain many bioactive constituents viz. flavanol, diterpene lactones, sesquiterpenes, ginkgolides, ascorbic acid, catechin, iron-based SOD, and p-hydroxybenzoic acid, are expected to have synergistic effects [205,206].

As inflammation and free radicals are suspected to possess a part in HD development, *G. biloba* is reported to possess antioxidant and anti-inflammatory characteristics [207,208,209]. Mahdy and colleagues found that *Ginkgo biloba* might repair some of the neurological problems caused by a toxin, 3-Nitropropionic acid (3-NP) [208]. When injected into the brains of mice, 3-NP mimics the effects of HD: it causes many of the biological and behavioral changes that are seen in people with HD. However, mice that were treated with both 3-NP and ginkgo biloba showed milder neurodegenerative problems than those treated with 3-NP alone. Several biochemical changes that occur upon exposure to 3-NP were mitigated in animals that were treated with *Ginkgo biloba*. Authors suggest that *Ginkgo biloba*’s antioxidant properties, antiapoptotic effects, and improvement of energy metabolism were responsible for the neuroprotective effects. The *G. biloba* extract improves the 3-NP induced neurobehavioral impairments [210] while also lowering striatal MDA levels. Glyceraldehyde-3-phosphate dehydrogenase and Bcl xl expression levels in the striatum are also down-regulated and up-regulated by standardized *G. biloba* extract (EGb 761). These biochemical findings, together with histological findings, suggested that EGb 761 can be utilized in HD [211].

### 8.5. Centella asiatica

It is popularly called Indian Pennywort, *Gotu kola*, and *Jal Brahmi*, and is a small, herbaceous, frost-tender perennial plant from the family Umbelliferae [212]. It is classified as a Rasayana in Ayurveda because of its quality to heal memory and age-related brain problems [213,214]. It is a controlling brain tonic that has long been utilized in Ayurvedic medicine to revitalize the body, boost intelligence, and treat cognitive problems such as Alzheimer’s disease [215,216].

Triterpenoid saponins, such as madecassoside, madecassic acid, asiaticoside, and asiatic acid (AA), are the key components of *C. asiatica* [217,218]. Research revealed the activity of AA on the neurodegenerative potential of *C. asiatica* in CNS directing on brain cells enhances the elongation of neuritis in an in vitro experimental model [219,220]. An in vivo study on *C. asiatica* leaf extract also demonstrated healing in the dendritic arborization of hippocampal CA3 neurons [221,222]. The mechanisms playing a putative role are MEK/ERK and PI3/Atk signalling pathways [223,224]. Moreover, the neurodegenerative effect of *C. asiatica* takes place via the MAP kinase pathway [219,223]. The most significant use of *C. asiatica* is regarded as a brain tonic to enhance memory function [225]. The ability to preserve mental function is attributed to its antioxidant characteristics. Various ROS scavenging experiments in vitro and in vivo have been established to discover this effect. *C. asiatica* improves faster functional recovery and enhanced axonal regeneration, according to a study [226]. An alternative study found a considerable elevation in dendritic length and branching sites in amygdaloid and hippocampal CA3 neurons [227]. As a result, the study’s findings suggest that *C. asiatica* has a possible protective action against any assault (caused by oxidative stress and mitochondrial damage), and memory-enhancing properties can help control HD and its consequences.

### 8.6. Xylaria Species

Xyloketal B is an extract obtained from marine mangrove fungus of *Xylaria* species. In the early phases of HD, the damage to brain cell connection arises in the areas that allow movement called the basal ganglia and the cortex [228]. Thus, figuring out how to prevent neuron mortality and increase the excitability of specific nerve cell connections could lead to new treatments. The identification of many unique, natural, and active chemicals often takes place in the marine environment [229]. Xyloketal B has established robust neuroprotection in contrasting models related to neuronal impairments [230,231]. Six xyloketal B derivatives were evaluated in a *Caenorhabditis elegans* HD model to find potent neuroprotective for HD; all six compounds demonstrated a preventive role [232]. The aromatic core structure of Xyloketal B features unique bicyclic acetal moieties that can be easily changed to ameliorate and broaden its activity [233,234]. Moreover, some xyloketal derivatives can form a hydrogen bond. Xyloketal adheres to mutant *htt* proteins and inhibits the *htt* aggregation process, hence slowing the progression of HD [235]. Molecular docking experiments demonstrate that it can bind to the mutant *htt* protein’s GLN369 and GLN393 residues, generating a stable trimeric complex that prevents mutant *htt* aggregation formation. Thus, xyloketal derivatives serve as novel drug candidates for treating HD [232].

## 9. Pain and HD

The increased CAG repeat in HD gene carriers leads to a gradual long polyglutamine repeat, which results in neuronal loss in the brain, most significant in the basal ganglia. The integration of motor, emotional, autonomic, and cognitive responses to pain are one of the fundamental functions of the basal ganglia in the processing and analgesia of pain. [236,237]. Compared to other patients’ symptoms, the pain might not seem like a major issue. Even still, it is misunderstood and poorly understood in HD, despite the fact that it might significantly improve the quality of life for those who are impacted. Numerous research has examined the root causes of chronic pain in HD patients. Muscle and endocrine dysfunction, which are putative sources of nociceptive and neuropathic pain in HD, may be exacerbated by mutant Huntingtin [238], perhaps through inflammatory and immune mechanisms [239]. A meta-analysis indicated that while the pain burden in HD was lower than that of the general population, the total mean prevalence of pain in HD was around 41% [240]. A cross-sectional analysis of the Enroll-HD study in carriers of the pre-manifest and manifest HD gene mutations and in carriers of the non-HD gene mutation was performed, which verified that the prevalence of pain interference was considerably higher in the middle stage of HD compared to not HD gene carriers and that the late and middle stages of HD had lower prevalence’s of painful situations [241]. According to reports, persistent pain is less common in HD patients who are evident, and it tends to get less common as the disease progresses. Patients with HD also experienced less severe pain and associated dysfunction. Additionally, with the complete phenotypical presentation of the disease in the middle and late stages, this characteristic, which was lacking in the pre-manifest phase, became relevant. Patients with more CAG expansion and decreased functional capacity appeared to have diminished pain perception [242].

## 10. Discussion

Huntington’s disease (HD) is a rare, neurodegenerative disorder characterized by chorea, behavioral manifestations, and dementia [243]. Although HD is rare, it does receive a great deal of research attention. One reason is that HD has some features that make it more likely to be a tractable problem than other neurodegenerative conditions. First, the autosomal dominant nature of the condition means that the diagnosis is it is possible to accurately model and study the disease in vitro and in vivo. It is estimated that the mean HD prevalence is 5 in 100,000 people. Moreover, in another study, it is estimated that one in every 10,000 persons-nearly, 30,000 in the United States, have Huntington’s disease. Juvenile Huntington’s occurs in approximately 16% of all cases [244,245]. Somatic instability of the CAG repeat occurs in the tissues that are most vulnerable to HD pathology, particularly the striatum, and the degree of instability negatively correlates with age at disease onset. Genetic association studies have shown that DNA repair components, particularly those involved in mismatch repair, modify somatic instability and disease course [117]. Mutation of *Htt* characterized with repeat expansion of CAG trinucleotides is the key factor in HD. Abnormal aggregation of mutant *Htt* protein may cause toxic effects in neurons, leading to a series of pathogenic mechanisms associated the alteration in proteostasis and protein degradation following mitochondrial dysfunction, oxidative stress, transcription and synaptic dysfunction, axonal transport impairment, and a series of metabolic impairments subsequent to neurodegeneration. Despite the fact that the pathogenesis of HD has still not been resolved and a cure is not available, many therapeutic options are available for treating symptoms and signs with a view to improving quality of life. Although many signs and symptoms can be treated, it is not always necessary to do so. To date, there are no promising treatments for the long-term unwanted effects of HD, which are being combated by symptomatic prevention and treatments for mitigating the psychiatric, cognitive, and motor deformities of HD. The patient’s limitations in daily life determine whether or not drugs are required. Very little evidence is available about the drug or the dosage to prescribe for any signs and symptoms. To overcome the above-mentioned concerns, investigation has been devoted to the isolation of novel compounds from a variety of natural products in modulating relevant neuro-degenerative disorders. Up until then, a plethora of traditional treatments based on natural products have been shown to possess a wide range of therapeutic benefits for HD under in vitro and in vivo models [246,247]. The neuroprotective effect of natural products in HD experimental models has been extensively studied. Indeed, based on relevant studies, natural products offer neuroprotection in experimental models predominantly through the antioxidant defense system, scavenging free radicals, neutralization of reactive oxygen species (ROS), reduction of oxidative stress, preservation of mitochondrial function, anti-inflammatory protection, inhibition of apoptosis, and induction of autophagy.

In future studies, it will be important to determine more precisely which components of the altered circuit contribute to deficits in learning, memory, and mood in the early stages because the development of more specific therapies for these symptoms would significantly improve the quality of life in affected individuals. Moreover, it will be critical to identify the earliest molecular mechanisms that lead to neuronal dysfunction and death in order to develop therapies that can delay the onset of overt HD.

## 11. Conclusions

HD is an inherited neurological illness in which *htt* is a protein that regulates transcription, transports intracellularly, and participates in the endosome-lysosome pathway. When this *htt* undergoes mutation, it causes several cellular complications viz., transcriptional dysregulation, protein aggregation and altered UPS, neuroglia dysfunction, mitochondrial dysfunction, altered synaptic plasticity, and axonal transport defect. This fatal disease is characterized by abnormal involuntary movements, impaired voluntary movements, and cognitive and psychiatric disturbances associated with neuronal death. Alternative and complementary therapies based on scientifically validated herbal ingredients may be an effective supplement to conventional medicine, which has potential drawbacks include the development of drug resistance and unpleasant side effects.

Naturally derived products having entrenched cell reinforcement and neuron safeguarding potential have indicated useful impacts against the manifestations of HD in both in vivo and in vitro studies. In this review, the roles of a number of plants are investigated in various neurotoxic animal models and transgenics are discussed, highlighting their ability to influence signalling pathways, leading to neuromodulation and probable neuroprotection. Moreover, the brief knowledge that the review provides on the pathologic mechanisms involved in HD are crucial points to consider while investigating therapeutic solutions and target specific relief. However, more investigation is needed to abundantly understand the potential therapeutic activity of phytochemicals in the prevention of HD. Hence, clinical outcomes of various studies are also needed to be evaluated in order to accept the efficacy of herbal medication in mainstream medicine.

## Figures and Tables

**Table 1:** Common symptoms of HD.

Common Symptoms of HD		References
**Motor symptoms**	Cerebellar Ataxia Chorea or choreoathetosis Rigidity Bradykinesia Dystonia Tremor Myoclonus Tics Gait Impairment and Falls Dysarthria Dysphagia	[52,53,54,55,56]
**Cognitive symptoms**	Deterioration of complex intellectual functions Impairment in the perception of time Impairment of spatial perception and unawareness Perseveration Impulsivity	[4,5,57,58]
**Psychiatric symptoms**	Depression Anxiety Irritability Agitation Clumsiness Altered sexuality Aggression Tendency to suicide Mania Delusions and Hallucinations Obsessions and Compulsions Apathy	[4,6,37,59,60,61,62]

**Table 2:** Stages of HD.

Developmental Stages of HD						
Characteristics	Early Stage 1	Early Intermediate Stage 2	Late Intermediate Stage 3	Early Advanced Stage 4	Advanced Stage 5	Reference
Duration	Continues from 0 to 8 years of disease onset.	Continues from 3 to 13 years of disease onset.	Continues from 5 to 16 years of disease onset.	Continues from 9 to 21 years of disease onset.	Continues between 11 and 26 years from disease onset.	[1,59,65]
Functions	Work, drive, handle money, and live independently.	Functional but lower work capacity.	Loss of workability, drive, mismanagement of finances, and household chores except eat, dress, and personal hygiene.	Dependent on extended care facility provided by the family.	Require support in all events of daily living.	[1,66]
Symptoms	Mild cognitive symptoms and psychiatric changes.	Chorea	Worsen of cognitive, psychiatric, and motor features.	Requires major assistance with basic functions (financial management, domestic responsibilities and living activities).	Difficulties with swallowing, communication, and weight loss.	[54,58,59,67]

## Abbreviations

HD	Huntington’s Disease
*Htt*	huntingtin
VEGF	vascular endothelial growth factor
TRP	tryptophan
KYN	Kynurenine
ANS	autonomic nervous system
CBP	CREB—binding protein
CREB	cAMP response element-binding
NRSE	Neuron restrictive silencer elements
CTD	carboxy-terminal domain
TBP	TATA-binding protein
HDACs	histone deacetylase complexes
UPS	ubiquitin-proteasome system
Hsp70	Heat-shock protein 70
HAP1	Huntingtin-associated protein1
ROS	reactive oxygen species
RNS	reactive nitrogen species
MDA	malondialdehyde
NO	nitric oxide
TGF-β	transforming growth factor beta
TLRs	Toll-like receptors
GABA_A_	γ-aminobutyric acid type A
AMPA	α-amino-3-hydroxy-5-methyl-4-isoxazole propionic acid
MSNs	Medium Spiny Neurons
